# A Primer Genetic Toolkit for Exploring Mitochondrial Biology and Disease Using Zebrafish

**DOI:** 10.3390/genes13081317

**Published:** 2022-07-23

**Authors:** Ankit Sabharwal, Jarryd M. Campbell, Tanya L. Schwab, Zachary WareJoncas, Mark D. Wishman, Hirotaka Ata, Wiebin Liu, Noriko Ichino, Danielle E. Hunter, Jake D. Bergren, Mark D. Urban, Rhianna M. Urban, Shannon R. Holmberg, Bibekananda Kar, Alex Cook, Yonghe Ding, Xiaolei Xu, Karl J. Clark, Stephen C. Ekker

**Affiliations:** 1 Department of Biochemistry and Molecular Biology, Mayo Clinic, Rochester, MN 55905, USA; sabharwal.ankit@mayo.edu (A.S.); jarrydmcampbell@gmail.com (J.M.C.); schwab.tanya@mayo.edu (T.L.S.); warejoncas.zachary@mayo.edu (Z.W.); mdwishman@msn.com (M.D.W.); hirotakaata@gmail.com (H.A.); wliu79@jh.edu (W.L.); n.ichino.gg@juntendo.ac.jp (N.I.); hunter.danielle@mayo.edu (D.E.H.); jdbergren@gmail.com (J.D.B.); urban.mark@mayo.edu (M.D.U.); urban.rhianna@mayo.edu (R.M.U.); holmberg.shannon@mayo.edu (S.R.H.); kar.bibekananda@mayo.edu (B.K.); alex.cook.school@gmail.com (A.C.); ding.yonghe@mayo.edu (Y.D.); xu.xiaolei@mayo.edu (X.X.); clark.karl@mayo.edu (K.J.C.); 2 Division of Cardiovascular Diseases, Department of Biochemistry and Molecular Biology, Mayo Clinic, Rochester, MN 55905, USA

**Keywords:** mitochondria, mitochondrial disorders, zebrafish, gene editing, TALEN, Gene Breaking Transposon

## Abstract

Mitochondria are a dynamic eukaryotic innovation that play diverse roles in biology and disease. The mitochondrial genome is remarkably conserved in all vertebrates, encoding the same 37-gene set and overall genomic structure, ranging from 16,596 base pairs (bp) in the teleost zebrafish (*Danio rerio*) to 16,569 bp in humans. Mitochondrial disorders are amongst the most prevalent inherited diseases, affecting roughly 1 in every 5000 individuals. Currently, few effective treatments exist for those with mitochondrial ailments, representing a major unmet patient need. Mitochondrial dysfunction is also a common component of a wide variety of other human illnesses, ranging from neurodegenerative disorders such as Huntington’s disease and Parkinson’s disease to autoimmune illnesses such as multiple sclerosis and rheumatoid arthritis. The electron transport chain (ETC) component of mitochondria is critical for mitochondrial biology and defects can lead to many mitochondrial disease symptoms. Here, we present a publicly available collection of genetic mutants created in highly conserved, nuclear-encoded mitochondrial genes in *Danio rerio*. The zebrafish system represents a potentially powerful new opportunity for the study of mitochondrial biology and disease due to the large number of orthologous genes shared with humans and the many advanced features of this model system, from genetics to imaging. This collection includes 15 mutant lines in 13 different genes created through locus-specific gene editing to induce frameshift or splice acceptor mutations, leading to predicted protein truncation during translation. Additionally, included are 11 lines created by the random insertion of the gene-breaking transposon (GBT) protein trap cassette. All these targeted mutant alleles truncate conserved domains of genes critical to the proper function of the ETC or genes that have been implicated in human mitochondrial disease. This collection is designed to accelerate the use of zebrafish to study many different aspects of mitochondrial function to widen our understanding of their role in biology and human disease.

## 1. Introduction

Mitochondria are semi-autonomous organelles critical for eukaryotic cell function. The mitochondrial endosymbiotic genesis hypothesis proposes its evolution from an alpha-proteobacterial ancestor, *Rickettsia prowazekii* [1,2], that was harnessed by a eukaryotic cell as the host billions of years ago [3]. The proteobacterium became a symbiote of the host cell, bringing with it a system for the more efficient generation of cellular energy in the form of ATP. The mitochondrial-encoded genetic material at present is a vestige of the original proteobacterial genome [4,5], meaning that despite having DNA of their own, mitochondria rely heavily on nuclear genes for most of their functions. Mitochondria have critical functions in metabolism, organ homeostasis, apoptosis, and aging. They also play important but still largely mysterious roles in human pathology, as demonstrated by the enormous biological variation and diverse disorders in patients with mitochondrial disease [6,7,8,9].

**Figure 1:**
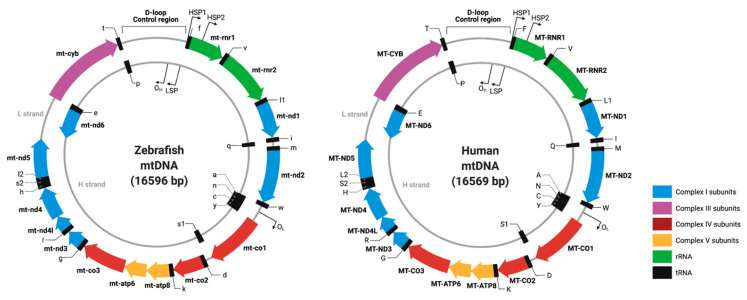
**Circular representation of the zebrafish and human mitochondrial genomes:** Both genomes share the same synteny, and the number of genes and are nearly identical in size.

Nearly every organ system can be compromised, but with highly variable and complex physiological and biochemical outcomes. The imaging and basic science of mitochondria showcase how this highly dynamic organelle responds differentially to extrinsic and intrinsic biological signals harboring a circular genome. The vertebrate mitochondrial chromosome is circular and includes 37 genes, 13 encoding for protein subunits of the electron transport chain, 22 coding for transfer RNAs, and 2 encoding ribosomal RNAs (Figure 1) [10,11]. The mitochondrial gene order, strand-specific nucleotide bias, and codon usage are highly conserved [12]. However, mtDNA-encoded genes lack introns and utilize a divergent genetic code compared to their nuclear counterparts [13,14]. Mitochondria are unique cellular compartments with different DNA and RNA repair and editing rules, hampering attempts to directly manipulate these nucleic acid components. For example, DNA nucleases that introduce double-stranded breaks and subsequent repair in nuclear DNA induce the degradation of the mtDNA [15,16,17]. Indeed, none of the canonical DNA repair pathways found in the nucleus are active in the mitochondria [16,18,19]. Finally, no system has demonstrated the ability to deliver exogenous DNA or RNA to mitochondria, restricting the tools available for the mtDNA editing [20,21]. However, understanding how mitochondria function in normal biology and how human mitochondrial DNA variations contribute to health and disease has been hampered by a lack of effective approaches to manipulating the powerhouse of the cell.

Recently, mtDNA editing has witnessed a fresh impetus with the arrival of programmable mitochondrial cytidine and deoxyadenosine deaminases, which have enabled the editing of mtDNA in cellular and model systems, though with sequence constraints for the pathogenic nucleotide residues to be targeted [22,23,24,25,26,27]. Unlike the nuclear genome, where most cells have only two copies, each cell can harbor thousands of copies of mtDNA, depending on environmental needs, making mitochondrial genome engineering a population genetics challenge. Clinical manifestations from either inherited or spontaneous mutations in these nuclear-encoded genes or in mtDNA lead to the alteration of the structure or function of the proteins or RNA that reside in the mitochondria. [7,8,28,29]. All these factors are distinct from the nuclear genome, making mtDNA a far less accessible genome for traditional gene-editing methods and reagents as compared to editing nuclear-encoded mitochondrial genes.

Discoveries from high-throughput proteomic approaches have enhanced our knowledge of the mitochondrial proteome [30,31,32]. The diverse functions that mitochondria are capable of, including oxidative phosphorylation, would not be possible with the small subset of 13 proteins encoded in mtDNA. There are approximately 1136 nuclear-encoded proteins that localize to the mammalian mitochondria, exerting dual genetic control via their nuclear counterpart [30]. Nuclear-encoded mitochondrial proteins based on their mitochondrial function can be broadly classified into different categories including oxidative phosphorylation, energy production, membrane dynamics, genome maintenance, and ion/metabolite homeostasis. Mitochondrial dysfunction tends to primarily affect high-energy systems and can therefore have devastating effects on a wide range of body systems, including brain function, liver function, vision, hearing, immune function, and all muscle types [29,33]. The identification and characterization of novel mitochondrial genes in human genetic disorders have also enhanced our knowledge of mitochondrial function [34,35,36,37,38].

The conserved roles of mitochondria and resident proteins have traditionally been uncovered using accessible model systems such as the yeast *Saccharomyces cerevisiae* [39,40,41] and invertebrates including *Caenorhabditis elegans* [42,43,44] and *Drosophila melanogaster* [45,46,47,48]. Vertebrate mitochondria, however, are known to have additional innovations and encode unique subunits not present in invertebrate models [4,49,50,51,52,53,54,55,56,57,58,59]. Most investigations of vertebrate mitochondrial biology to date have been conducted either in cell culture or in mouse models. However, cells in a dish are missing important environmental cellular and organismal contexts, whereas mouse mitochondrial experimental work can be hindered by limited population size and invasive imaging techniques.

We provide here an initial genetic toolkit for modeling mitochondrial biology and disease in *Danio rerio* (zebrafish) to help catalyze the use of this invaluable model organism in mitochondrial research. The zebrafish is a tropical freshwater teleost that has proven to be an invaluable resource for the study of human disease and genetics [60,61,62]. Principal among the many research-amenable characteristics of zebrafish is their high genetic orthology to humans, high fecundity, their optical transparency (in the embryonic stage) that lends itself to facile imaging, and the ease of reagent delivery through microinjection into a single-celled embryo, which is a full millimeter across before the first mitotic division. Zebrafish also serve as a potentially powerful vertebrate model organism to study human mitochondrial disorders because of the conserved mitochondrial genome and mitochondrial genetic machinery. Zebrafish and human mitochondrial chromosomes display ~65% sequence identity at the nucleotide level and share the same codon usage, strand-specific nucleotide bias, and gene order (Figure 1) [10].

Over recent years, zebrafish research has helped shed light on mitochondrial biology and further developed our understanding of the mechanisms of mitochondrial-associated pathology with phenotypes pertaining to cardiac, and neural organogenesis to name a few [63,64]. Zebrafish have also been successfully used as a model system to study mitochondria-targeting drugs with implications for development and cardiovascular function [65,66,67]. These drugs can simply be dissolved in the water housing the larvae and offer the advantage of a large sample size with minimal volumes of the drug administered. Drug-screening studies have aided in understanding the pathogenesis of various diseases and helped to identify targets for treatment.

To test hypoxia as a potential protective therapy in mitochondrial disorders, Von Hippel-Lindau (*vhl*)-null mutant zebrafish, when treated with antimycin-mediated mitochondrial insult, exhibited improved survival. In addition, FG-4592 was found to improve survival in response to respiratory chain inhibition, possibly due to an increase in hypoxia response [68]. A series of ETC complex-specific pharmacological inhibitors such as rotenone (complex I), azide (complex IV), oligomycin (complex V) and chloramphenicol (mitochondrial protein translation) have been used to model respiratory chain dysfunction in zebrafish. Zebrafish larvae display a series of phenotypes, such as developmental arrest, when treated with rotenone. Treatment with azide induced decreased heart rate, loss of motor function, inability to respond to tactical stimulation, neurological damage, and mortality [67,69].

Zebrafish offer many unique advantages for in vivo imaging experiments, as compared to their mammalian counterparts. A classical study corroborating this advantage is the in vivo imaging of mitochondrial transport in a single axon, as demonstrated by Yang and colleagues [70]. Seok and colleagues generated a transgenic zebrafish line expressing green fluorescent protein fused to a mitochondrial localization sequence from cytochrome c oxidase [65]. Mitochondrial function is often measured by various parameters such as the estimation of membrane potential, mitochondrial superoxide species, and energy production. Superoxide activity and membrane potential in zebrafish have been measured by employing the use of cell-permeable chemical probes such as MitoSox [71] and Dihydrorhodamine 123 (DH123) [72], respectively. Zebrafish transgenic lines expressing genetically encoded calcium and oxidation indicators have also served as an excellent model to measure calcium homeostasis and oxidative status in vivo in models of mechanosensory hair cell damage and death [73]. Constructs such as Mitotimer allow the measurement of mitochondrial turnover, transport, and changes in the redox history of mitochondria during organogenesis events in zebrafish embryos [73]. This transgenic model enables a sweeping picture of the mitochondrial network, helping in the study of cellular processes such as mitophagy and apoptosis. Zebrafish have also provided novel mechanistic insights to interrogate the mitochondrial dynamics, called the “in vivo life cycle of mitochondria” in healthy and diseased conditions [74]. One such example is that of Mitofish [75], which recapitulates mitochondrial network biogenesis, unraveling the role of this organelle in cell-type-specific niches of different organ systems. Mitofish is a transgenic zebrafish line that fluorescently labels the mitochondria in the neurons, enabling non-invasive in vivo observation. Advancements in adaptive optics and lattice light-sheet microscopy have empowered researchers to look at vibrant and colorful images of organelles in zebrafish. These technologies have been applied to investigate the organellar dynamics in the brain during early development and in the eye of an adult zebrafish [63,76].

To help engender an expansion of zebrafish deployment in mitochondrial research, we present a panel or “starter-pack” toolkit of genetic mutants made in nuclear-encoded mitochondrial genes in zebrafish. This mutant panel, which we have named the Marriott Mitochondrial Collection (MMC), consists of 26 zebrafish lines with mutations in 23 different mitochondrial genes. The mutant collection focuses primarily on genes that encode components of the energy-generating electron transport chain with at least one mutant in each complex of the ETC. Other mutants consist of assembly factors, protein chaperones that manage mitochondrial membrane traffic, and genes related to mitochondrial replication. These mutants were made either using targeted endonuclease [77] or curated from our research group’s library of randomly generated insertional mutants [78,79]. We hope that this collection, in addition to being intrinsically useful, will also help serve as a primer for the modeling of mitochondrial biology and disease in zebrafish.

## 2. Methods

### 2.1. Zebrafish Handling

All animal work was conducted under Mayo Clinic’s institutional animal welfare approvals (IACUC number: A34513-13-R16).

### 2.2. Identification of Zebrafish Orthologs Having a Putative Mitochondrial Function

A previous study combining discovery and subtractive proteomics with computational microscopy identified 1140 mouse genes that could encode for proteins residing in the mitochondria [30]. They further identified 1136 human orthologs for these genes, providing an initial inventory of the genes coding for proteins resident in mitochondria. Using literature assessment and HUGO database curation approaches, we identified 97 proteins that are involved in the biogenesis and assembly of electron transport chains in mitochondria. Using zebrafish orthologs of human genes from ZFIN (Zebrafish Information Network), 92 zebrafish mitochondrial orthologs were identified. These orthologs were systemically annotated with respect to clinical phenotype by extensive mining from PubMed-based published case reports and the OMIM database (Supplementary File S1).

### 2.3. Mutant Generation

Mutant lines were created by one of two methods either through the targeted use of Transcription Activator Like Effector Nucleases (TALENs) [77] or by screening Gene Breaking Transposon (GBT) lines for integrations into mitochondrial genes [78,79]. Reagents were delivered in both methods by the microinjection of either TALEN pairs or GBT transposon and Tol2 transposase into single-cell zebrafish embryos.

### 2.4. TALEN Design/Assembly/Delivery

The TALEN mutants in this collection were originally generated as part of a previously published study [77]. In brief, TALEN pairs were designed [77] using the Mojo Hand software platform [80] (www.talendesign.org) to target highly conserved, and therefore likely functionally important, genetic loci of nuclear-encoded mitochondrial genes. TALEN RVDs were then cloned into pT3Ts-GoldyTALEN (TALEN vector with a T3 transcriptional promoter for in vitro transcription) using the FusX rapid TALEN assembly system [77], which uses the RVD definitions: HD = C, NN = G, NI = A, NG = T. Following assembly, mRNA was synthesized in vitro using the mMessage mMachine T3 kit (Ambion) and extracted by a phenol-chloroform extraction, as prescribed in the mMessage mMachine manual. The extracted mRNA was then delivered into single-cell zebrafish embryos at 100 pg doses (50 pg per TALEN arm) by microinjection.

### 2.5. TALEN Mutant Screening

Following microinjection, genomic DNA was extracted from F0 larvae three days post-fertilization (dpf) by sodium hydroxide extraction. DNA was analyzed for NHEJ activity at the TALEN target site for eight individual larvae by Restriction Fragment Length Polymorphism (RFLP) analysis. Groups with high reported NHEJ activity by RFLP were raised to adulthood and outcrossed to create an F1 generation. Suspected NHEJ mutants were further verified by Sanger sequencing. F1 larvae demonstrating NHEJ mutations confirmed by both RFLP and Sanger sequencing were raised to adulthood. Fin biopsies were performed on these adults and DNA was extracted by sodium hydroxide extraction.

### 2.6. Gene Breaking Transposon System

The Gene Breaking Transposon System of protein trap system and a complete repository of protocols for the creation and screening of GBT mutant lines have been described [78,79]. In short, protein trap transposons were delivered in combination with mRNA for Tol2 transposase (25 pg each) into single-cell zebrafish embryos by microinjection. Embryos were screened for GFP fluorescence at 3–4 dpf. Embryos, those with whole-body GFP expression, were raised to adulthood and outcrossed to non-transgenic lines to create an F1 generation. mRFP-expressing F1 embryos were sorted by expression pattern, assigned a GBT number, and raised to adulthood. These adult fish were then outcrossed to non-transgenic lines to create an F2 generation upon which all subsequent propagation, testing, and imaging were conducted. To determine genes tagged by the protein trap system, the rapid amplification of cDNA ends (RACE) was performed as described in [78] with minor updates to primer sequences. cDNA was generated using a transposon-specific primer (5R-mRFP-P0) against 250 ng of total mRNA in the reverse transcription reaction. PCR was then performed using the following gene-specific primers: 5R-mRFP-P1 and 5R-mRFP-P2. The resulting products were TA cloned for further amplification and then sequence-verified for in-frame mRFP fusions by Sanger sequencing. In some cases, inverse PCR was also conducted to RACE PCR as described [78]. Whole genomic DNA was extracted from individual F2 embryos using a sodium hydroxide extraction and 800 ng was digested in a combination reaction using AvrII, NheI, SpeI, and XbaI restriction enzymes. Approximately 200 ng of the product of this digestion was self-ligated and used as a template for PCR using the following gene-specific nested and primary primers. At the 5’ side: 5R-mRFP- P1 and 5R-mRFP-P2 paired with INV-OPT-P1 and INV-OPT-P2, respectively; 3’ side: 5R-GFP- P1 and 5R-GFP-P2 with Tol2-ITR(L)-O1 and Tol2-ITR(L)-O3, respectively. The products of the final nested reaction were gel-extracted, cloned, and sequenced. Prospective in-frame mRFP fusions were further verified by comparing the suspected protein trap allele to GBT expression patterns and by PCR against DNA or cDNA from mRFP carrier siblings versus non-carrier siblings. As verified by these methods, protein trap lines were cataloged for specific genomic fusions using the National Center for Biotechnology Information’s (NCBI) HomoloGene database. Human orthologs for tagged genes were further identified using blastX searches against the human genome. GBT lines with protein trap fusions to nuclear-encoded mitochondrial genes with human orthologues have been included in this collection.

### 2.7. In Silico Analysis to Determine Protein Homology and Alteration of DNA Sequence by TALENs and the GBT in Zebrafish Mutants

Human amino acid sequences were compared to both the zebrafish wild-type sequence and each specific zebrafish mutant sequence associated with each allele. For humans, amino acid sequence information for a particular gene was gathered from https://www.uniprot.org/ (accessed on 26 January 2015, 4 June 2017) along with the wild-type amino acid sequence for the zebrafish. The first/most common isoform for each entry was used for this analysis. Any added information regarding functional domains or regions was also gathered. A protein-protein BLAST was conducted using https://blast.ncbi.nlm.nih.gov/Blast.cgi (accessed on 26 January 2015, 4 June 2017) to compare the sequences of the human and wild-type zebrafish amino acid sequences. The BLAST search settings used included the blastp algorithm. Regions of low and high homology were then mapped out for the sequences. For zebrafish mutant to human comparisons, the cDNA or DNA sequences were gathered via sequencing and put through the translate tool at https://web.expasy.org/translate/ (accessed on 26 January 2015, 4 June 2017). The standard genetic code was used for conversion. Stop codons arising from the frameshifted DNA sequence were found in all mutants. The BLAST analysis conducted on the mutants was taken from the starting methionine to the first encountered stop codon. Regions of low and high homology were then mapped for each sequence.

### 2.8. RNA Extraction and Sample Preparation

Adult zebrafish mutants were incrossed and larvae were genotyped at 6 dpf. RNA isolation was performed using TRIzol extraction. As part of the study design, 6 dpf individual zebrafish embryos were pooled together for RNA extraction. This approach was based on a previously published gene expression study [81], wherein pooling of samples has been shown to reduce variance where each sample can be referred to as an internal biological replicate. Pooled zebrafish larvae were homogenized in 500 µL TRIzol, using a handheld homogenizer, and the lysate was incubated at room temperature for 5 min. Following this, 140 µL of chloroform was added to the homogenate and was incubated on ice for 15 min. The reaction mixture was then centrifuged in a benchtop centrifuge at 4 °C for 15 min. After the phase separation, the aqueous layer was removed and placed in a separate 1.7 mL centrifuge tube. The aqueous layer was put through purification steps of ethanol and DNase I treatment to obtain pure RNA. Quantity and integrity of RNA were measured using a spectrophotometer and agarose gel electrophoresis before the submission for Genewiz RNA-sequencing.

### 2.9. RNA Sequencing and Analysis

RNA library preparations and sequencing reactions were performed at GENEWIZ, LLC. (USA). Quality-checked samples were then prioritized for making libraries for RNA sequencing using the NEB Next Ultra RNA Library Prep Kit for Illumina using the manufacturer’s instructions (NEB, USA). Paired-end reads of 150 bp length were generated for the mutants and wild-type samples. Phred score of Q30 was applied as a base quality cut-off to trim raw reads using Trimmomatic [82]. Reads that passed the threshold quality score of Q30 were then pseudoaligned to the zebrafish reference genome Zv10 version. Transcript assembly and calculation of relative expression values across transcripts were performed using Kallisto [83]. Transcript level counts at the gene level were summarized using Tximport [84]. Differential expression was computed using DESeq2 as counts per million (CPM) [84]. Fold change cut-offs of ≥log_2_1 and ≤log_2_−1 were applied to prioritize the genes to be considered as upregulated and downregulated, respectively. Gene set enrichment analysis (GSEA) was performed using a web-based integrated analysis platform Genetrail3 [85]. Each of the gene obtained from the RNA sequencing analysis was assigned a score as per a defined formula (Score- [−log_10_(*p*-value) × log_2_fold change]). Genes with respective scores assigned to them were fed as the input file to obtain the list of enriched/depleted biological pathways from the Reactome, Wiki and KEGG pathways. Kolmogorov–Smirnov test was used as the statistical test for the GSEA analysis. PANTHER database [86] was used to identify the biological process in which the differentially expressed genes are involved, as well as the protein class to which they belong.

### 2.10. Access to All Reported Reagents—Zebrafish and Sequences

All listed tools are immediately available through the Mayo Clinic Zebrafish Facility, and all fish lines will be available via ZIRC. Sequences needed for genotyping and related metadata are currently on zfishbook [87].

## 3. Results

### 3.1. Generation of Zebrafish Mutant Collection

**Figure 2:**
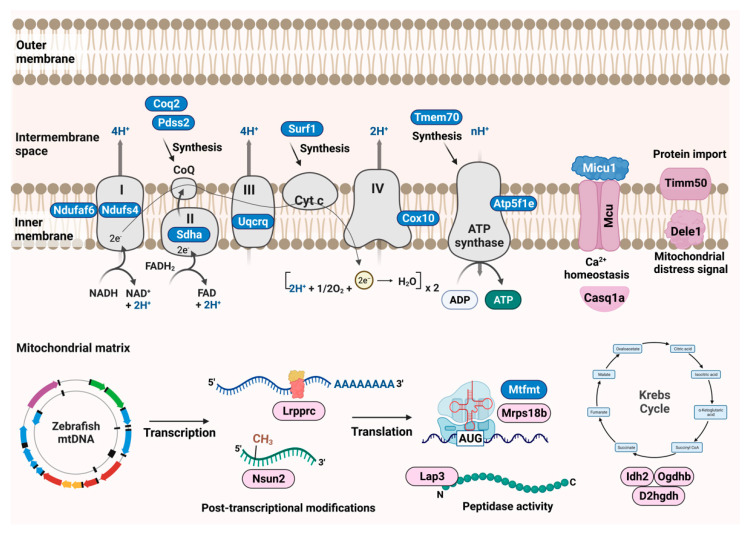
**Zebrafish Marriott mitochondrial mutant collection:** Schematic representation of various mitochondrial resident pathways for which zebrafish mutants were generated. The nuclear-encoded mitochondrial proteins have been illustrated according to the function they are involved in mitochondrial maintenance and homeostasis. Mutants generated by TALE gene editing are depicted as blue, whereas those generated by the GBT system are depicted in pink.

We generated zebrafish mutants for a wide selection of nuclear-encoded mitochondrial genes by employing two genetic engineering approaches, gene-breaking transposons, and TALENs. The collection of zebrafish mutants is referred to as the Marriott Mitochondrial Collection (MMC) and comprises 26 mutants for 23 nuclear-encoded mitochondrial proteins (Figure 2). Out of these, 15 were created by TALEN indel mutagenesis and 11 were created by gene-breaking trap mutagenesis. The mutants include proteins from known functional pathways involved in mitochondrial homeostasis and ATP generation (Figure 2).

Broadly, the pathways can be classified as subunits of oxidative phosphorylation complexes, chaperones for the assembly of oxidative phosphorylation proteins, and maintenance proteins for the mitochondrial genome (replication, mtRNA folding, and translation), calcium homeostasis, and mitochondrial protein import. All of these genes have human orthologs, nearly all of which have known mutations that lead to severe clinical manifestations such as Leigh syndrome, cardiomyopathy, progressive external ophthalmoplegia, and oxidative phosphorylation deficiency (Table 1).

### 3.2. TALEN- and GBT-Mediated Targeting of Nuclear-Encoded Mitochondrial Genes

**Figure 3:**
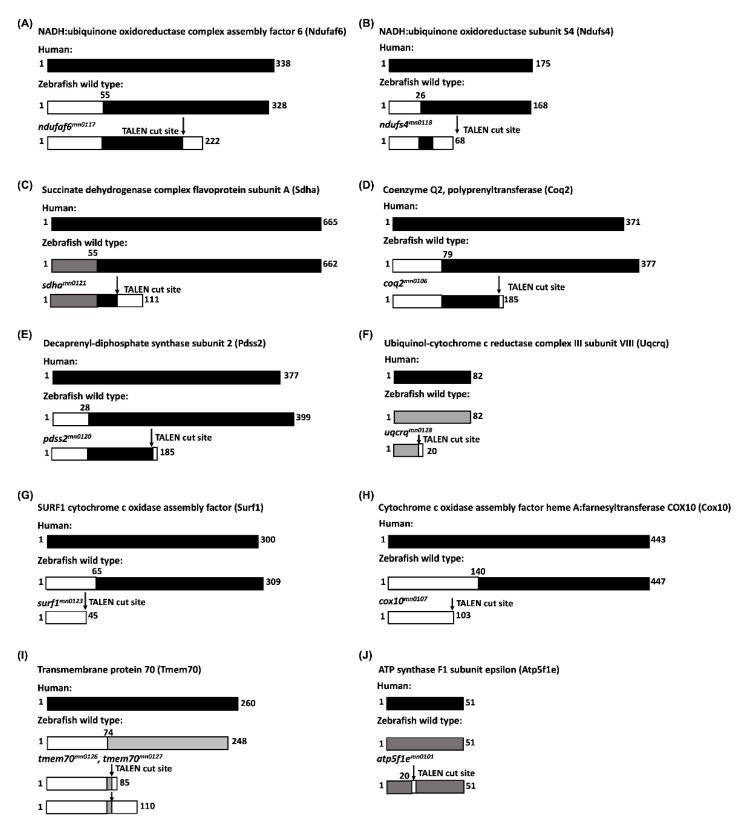

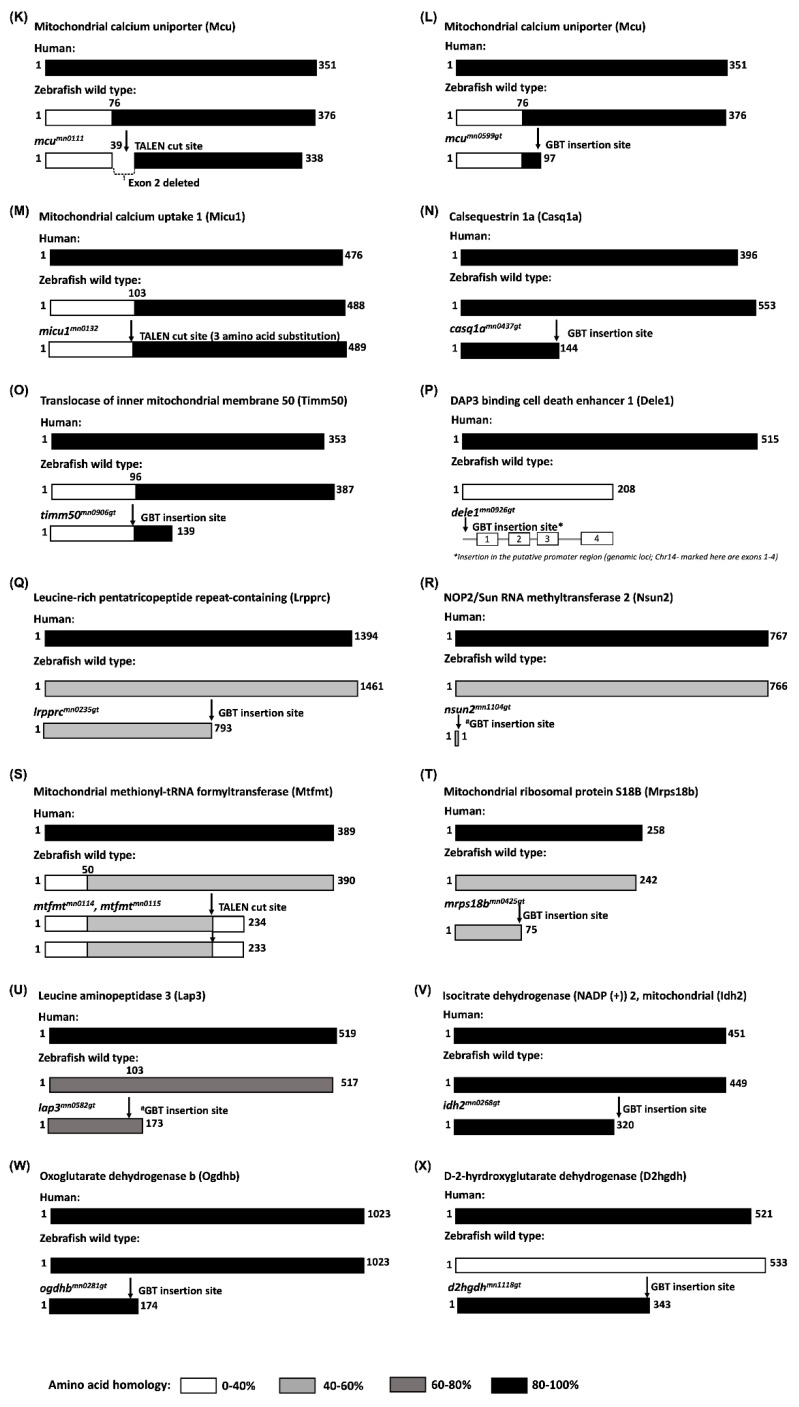
A depiction of homology of the mutants created for proteins involved in different mitochondrial resident pathways: (**A**,**B**): Complex 1 of mitochondrial respiratory chain; (**A**): NADH: ubiquinone oxidoreductase complex assembly factor 6 (Ndufaf6); (**B**): NADH: ubiquinone oxidoreductase subunit S4 (Ndufs4); (**C**): Complex 2 of mitochondrial respiratory chain; (**C**): Succinate dehydrogenase complex flavoprotein subunit A (Sdha); D-E: Biosynthesis of coenzyme Q; (**D**): Coenzyme Q2, polyprenyltransferase (Coq2) (**E**): Decaprenyl-diphosphate synthase subunit 2 (Pdss2); (**F**): Complex 3 of mitochondrial respiratory chain; (**F**): Ubiquinol–cytochrome c reductase complex III subunit VIII (Uqcrq); (**G**,**H**): Complex 4 of mitochondrial respiratory chain; (**G**): SURF1 cytochrome C oxidase assembly factor (Surf1); (**H**): Cytochrome C oxidase assembly factor heme A:farnesyltransferase COX10 (Cox10); (**I**,**J**): Complex 5 of mitochondrial respiratory chain; **(I)**: Transmembrane protein 70 (Tmem70); (**J**): ATP synthase F1 subunit epsilon (Atp5f1e); **(K**–**N)**: Mitochondrial calcium homeostasis; (**K**): Mitochondrial calcium uniporter (Mcu) generated by TALEN and (**L**); by GBT; (**M**): Mitochondrial calcium uptake 1 (Micu1); (**N**): Calsequestrin 1 (Casq1a); (**O**): Mitochondrial protein import; (**O**): Translocase of inner mitochondrial membrane 50 (Timm50); (**P**): Stress response; (**P**): DAP3 binding cell death enhancer 1 (Dele1) (GBT insertion site is in the putative promoter region, site is depicted upstream of exons of *dele1* gene in chromosome 14; (**Q**–**U**): Mitochondrial genome maintenance; (**Q**): Leucine-rich pentatricopeptide repeat containing (Lrpprc); (**R):** NOP2/Sun RNA methyltransferase 2 (Nsun2) ^#^*candidate line*; (**S**): Mitochondrial methionyl-tRNA formyltransferase (Mtfmt); (**T**): Mitochondrial ribosomal protein S18B (Mrps18b); (**U**): Leucine aminopeptidase 3 (Lap3) ^#^candidate line; (**V**–**X**): Mitochondrial metabolite synthesis; (**V**): Isocitrate dehydrogenase (NADP(+)) 2, mitochondrial (Idh2); (**W**): Oxoglutarate dehydrogenase b (Ogdhb); (**X**): D-2-hydroxyglutarate dehydrogenase (D2hgdh).

Amino acid analysis using protein–protein BLAST functions between the wild-type human, wild-type zebrafish, and mutant zebrafish sequences showed consistent results across the two species and mutant and wild-type genotypes. The comparison of wild-type human and wild-type zebrafish sequences showed high areas of homology following the mitochondrial targeting domain in almost every gene, with 80–100% similarity in the catalytic or active domains of the transcripts. The analysis of the mutant zebrafish and human wild-type comparison showed a range of differences with predicted frameshift mutations leading to the truncation of the protein very early in the transcript (Figure 3A–X). Of the alleles created by TALEN mutagenesis in the MMC collection, all but one (*micu1)* showed a predicted frameshift mutation, leading to a truncation event immediately following the NHEJ-mediated insertion or deletion. The GBT mutants showed high levels of homology prior to the transposon integration site followed by a nearly complete loss of normal transcript levels the following splicing into the GBT cassette.

### 3.3. Mitochondrial Zebrafish Mutants Display Altered Transcriptomic Signature

To assess the genome-wide transcriptional alterations in a sampled subset of the Marriott mitochondrial mutants, strand-specific paired-end RNA sequencing was conducted to develop a candidate differential transcriptional signature. From the collection, the following mutants were prioritized for the transcriptomic analysis *coq2*, *uqcrq,* and *mcu*. Reads in the range of 66–99 million were generated for *coq2*, *uqcrq*, *mcu* mutants, and wild-type controls. Low-quality reads with a Phred score cutoff value of Q30 were filtered and threshold qualified reads were then aligned onto the Zv10 zebrafish reference genome. Reads were pseudoaligned with an average mapping percentage of ~96% and approximately 76% of the reads were uniquely aligned to the zebrafish genome. Uniquely mapped reads were then assembled across the genome to quantify the expression of transcripts. The empirical cutoffs of genes with log_2_fold change ≥1 and log_2_fold change ≤−1 were shortlisted as upregulated and downregulated, respectively, in the mitochondrial zebrafish mutants.

**Figure 4:**
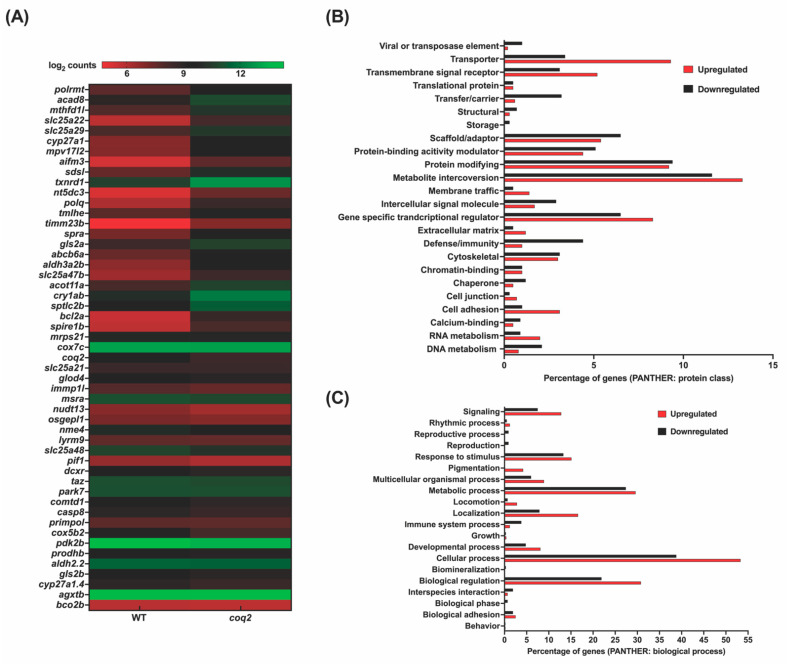
**RNAseq analysis of *coq2* mutants:** (**A**)**:** Heat map visualization of expression of zebrafish orthologs for human MitoCarta genes. The gradient color scale represents the log_2_CPM value obtained for each of the zebrafish mitochondrial orthologs in the two datasets. (**B**,**C**): PANTHER classification for all the differentially expressed genes in the homozygous mutant according to protein class (**B**) and biological process (**C**). Each histogram represents the percentage of genes falling in each of the categories.

For the *coq2* mutants, 1316 and 848 genes were observed to be upregulated and downregulated, respectively (Supplementary Figure S1 and File S2). From this set, 24 and 27 human orthologs of upregulated and downregulated zebrafish genes, respectively, overlapped with the human MitoCarta3.0 database (Figure 4A). Differentially expressed genes were then classified using the PANTHER classification system (Protein ANalysis THrough Evolutionary Relationships) and binned as per protein class (Figure 4B) and biological process (Figure 4C). The majority of the differentially expressed ones are encoded for proteins performing functions such as metabolite interconversion and gene-specific transcriptional regulators. As *coq2* is a key mitochondrial electron transport chain protein, we expected to observe the metabolic process and cellular process being altered, as evident from the genes belonging to these classes. *coq2* zebrafish mutants exhibited altered expressions of key mitochondrial genes such as cytochrome c oxidase subunit 5B2 (*cox5b2)* and cytochrome c oxidase subunit 7C *(cox7c)*. Genes pertaining to mitochondrial genome and membrane maintenance such as polymerase (RNA) mitochondrial (DNA directed) (*polrmt)*, MPV17 mitochondrial inner membrane protein-like 2 (*mpv17l2)*, and solute carrier family 25 member 21 (*slc25a21)* were also observed to be differentially expressed in these mutants. These findings were further corroborated by the gene set enrichment analysis where, for example, the mitochondrial ribosomal biogenesis and respiratory chain pathways were observed to be depleted.

**Figure 5:**
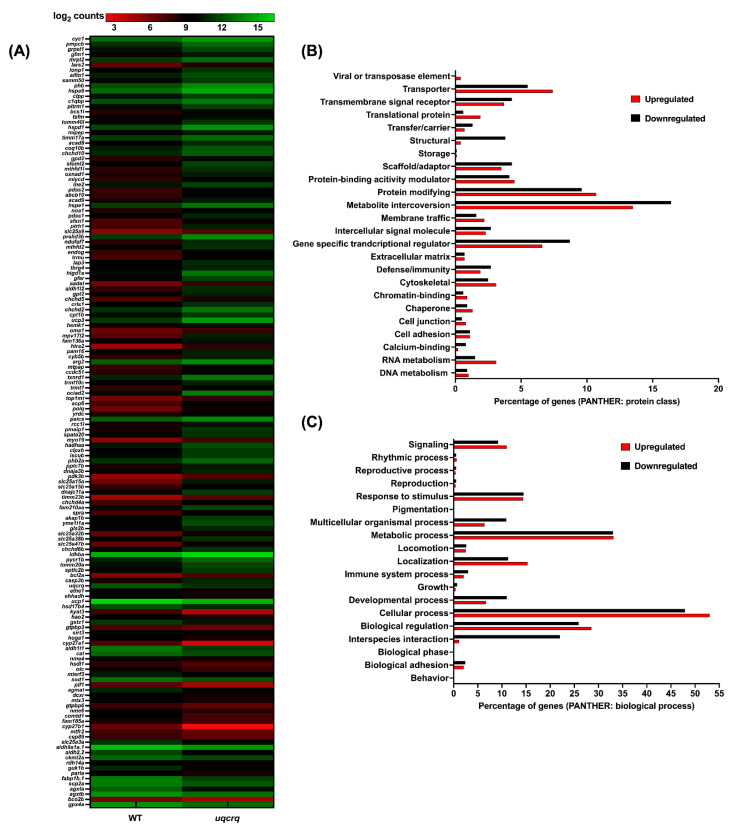
**RNAseq analysis of *uqcrq* mutants:** (**A**): Differential expression of zebrafish orthologs for human mitochondrial resident proteins; MitoCarta is represented as a heat map. The gradient color scale represents the log_2_CPM value obtained for each of the zebrafish mitochondrial orthologs in the two datasets. (**B**,**C**): Differentially expressed genes classified according to protein class (**B**) and biological processes (**C**). Each histogram represents the percentage of genes falling in each of the categories.

In the case of *uqcrq* mutants, the bioinformatic analysis revealed that 1744 genes were upregulated, and 1370 genes were downregulated for the *uqcrq* loss of function zebrafish model (Supplementary Materials Figure S1 and File S3). Overall, 105 and 143 upregulated and downregulated zebrafish orthologs were identified for mitochondrial resident human proteins (Figure 5A). *uqcrq* mutants also displayed a similar trend to the *coq2* counterparts, wherein the majority of the differentially expressed genes belonged to protein-modifying, gene-specific transcriptional regulator, and metabolite interconversion categories (Figure 5B). Biological processes that were majorly represented by these genes were cellular processes, metabolic processes, and biological regulation. Interestingly, mutants exhibiting a loss of function in *uqcrq* displayed enrichment of pathways related to mitochondrial bioenergetics and ribosome biogenesis.

**Figure 6:**
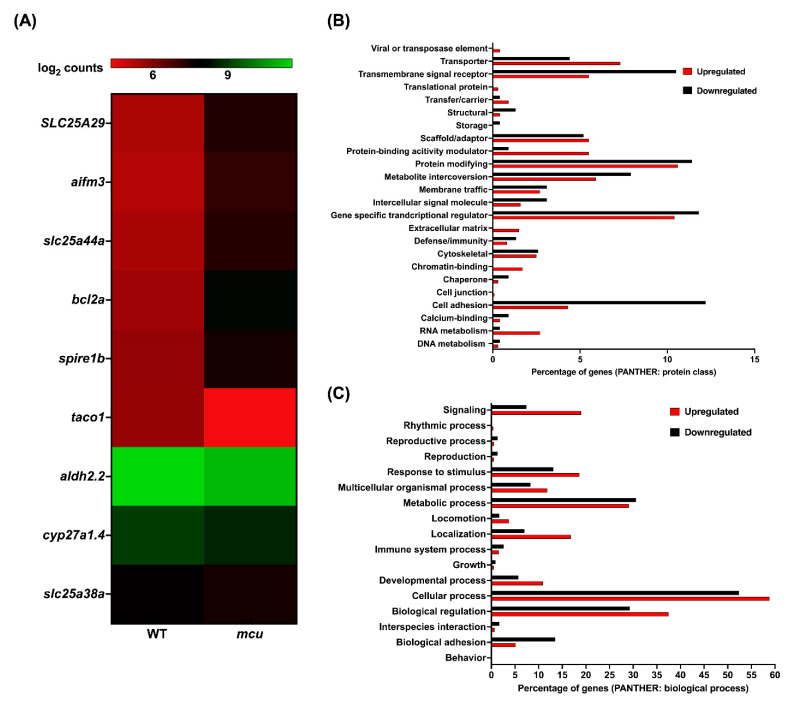
**RNAseq analysis of *mcu* mutants:** (**A**): Heat map representation of zebrafish orthologs for human mitochondrial localized proteins (MitoCarta). The gradient color scale represents the log_2_CPM value obtained for each of the zebrafish mitochondrial orthologs in the two datasets. (**B**,**C**): Classification by PANTHER system for all the differentially expressed genes in the homozygous mutant according to protein class (**B**) and biological process (**C**). Each histogram represents the percentage of genes falling in each of the categories.

A total of 937 genes were observed to be upregulated and 200 genes were downregulated in the *mcu* mutants (Supplementary Materials Figure S1 and File S4). Overall, five and four human orthologs of zebrafish upregulated and downregulated genes aligned with the MitoCarta 3.0 inventory (Figure 6A). Gene set enrichment analysis using the GeneTrail predicted pathways pertaining to mitochondrial ribosome biogenesis, and mitochondrial respiration depleted in these mutants. The downregulation of genes belonging to the SLC25 family was observed in *mcu* zebrafish mutants harboring a 39bp in-frame deletion. Most of the differentially expressed genes were in the protein-modifying, gene-specific transcription regulators, and cell adhesion categories (Figure 6B). Biological processes that were represented by the differential genes were predominantly cellular processes, biological regulation, and metabolic processes.

## 4. Discussion

In a multicellular organism, each cell is able to carry out its functions due to the well-orchestrated cross-talk between the nuclear and mitochondrial genomes. Many mitochondrial functions such as energy production, genome maintenance, ion/metabolite homeostasis, membrane dynamics, and the transport of biomolecules can be attributed to approximately 1136 known nuclear proteins residing in the mitochondria [30]. Due to advancements in proteomic technologies in recent years, there has been a surge in the documentation of many characterized and uncharacterized mitochondrial proteins. However, the correlation between mitochondrial localization of these proteins and their physiological significance in disease progression remains largely unexplored. Out of the 1136 nuclear-encoded proteins, only ~25% of the genes have functional evidence of mitochondrial clinical manifestations [33]. For the remaining ~75% of the proteins, mitochondrial involvement in disease progression has yet to be demonstrated. The obstacles in mitochondrial genetic research, and thus delays in finding effective treatments, are primarily due to the limited tools available to mitochondrial researchers, specifically the small number of available model systems and animals.

Most mitochondrial research thus far has made use of model systems that each present their unique challenge to the accurate study of human disease. Yeast, one of the most common laboratory eukaryotes, has been incredibly useful in mitochondrial research, but unlike humans and other animals, they lack complex I [49]. A complex 1 deficiency is the most frequent metabolic phenotype among mitochondrial disorders caused by mutations in 28 out of 48 genes, contributing to its assembly and biogenesis [88]. Human cell culture has been valuable in exploring mitochondrial disease models, principally through cytoplasmic hybrids that are made by replacing the mitochondria of an immortalized cell line with mutated patient mitochondria [89,90,91]. However, while these cells are a more faithful representation of human mitochondrial activity than yeast, they have different barriers to accuracy. First, immortalized cells, and cultured cells in general, often have modified metabolic pathways compared to cells in vivo. Second, as is common in all cell culture research, cells in a dish cannot accurately represent the complex interactions and systems biology inherent to a complete organism.

In this study, we aim to support the establishment of the zebrafish as a complementary animal model for understanding the role of nuclear-encoded mitochondrial proteins in biology and the pathophysiology of mitochondrial disorders. We propose that zebrafish can be an excellent model organism to study mitochondrial biology primarily because of their conserved genome, codon bias, and synteny. Other advantages include their amenability to genetic manipulation and optical clarity, which facilitates direct observation. New gene-editing techniques such as TALENs and CRISPRs have aided in the development of humanized disease models in zebrafish [92,93].

The MMC collection described here encompasses mutations in 23 different nuclear-encoded genes related to mitochondrial function, enabling a diverse study of the important roles that mitochondria play in cellular biology. The collection includes mutants in the complexes of the electron transport chain as well as many other crucial pathways related to protein transport, metabolite synthesis, mtDNA gene expression and translation, and calcium homeostasis. The first set of mutants was created using custom gene editing to exons coding for critical functional domains of the protein product. These domains were targeted because they shared high levels of homology to their human ortholog. The second group of mutants consisted of fish that had been injected with a protein trap transposon system that truncates the expected protein products as well as sorting and imaging through fluorescent expression patterns in vivo. Included here, we curated a small group of mutants where the GBT is integrated into a mitochondrial gene of interest. In recent years, zebrafish have been used to understand the pathophysiology of human mitochondrial disorders such as cardiovascular, multisystemic, neurological, and erythropoiesis disorders. Taking cues from these studies, many of the genes selected here were prioritized based on their role in the pathophysiology of nuclear-encoded mitochondrial disorders.

Whole-embryo RNA sequencing was adopted to identify the differential expression of genes encoding for the proteins responsible for orchestrating the mitochondrial function. We conducted a preliminary transcriptomic analysis on the prioritized three mutant lines from the collection. These zebrafish mutants harbored edits in the genes *coq2*, *uqcrq,* and *mcu*. Coq2 protein is involved in the biosynthesis of CoQ, a redox carrier in the mitochondrial chain. *coq2* zebrafish mutants exhibited an altered expression of key mitochondrial genes such as cytochrome c oxidase subunit 5B2 (*cox5b2)* and cytochrome c oxidase subunit 7C *(cox7c)*. The overlapping transcriptomic signatures observed across all these mitochondrial mutants was due to an alteration of pathways related to oxidative phosphorylation and translation, which were consistent with one of the characterized models from the MMC collection that harbors a disruptive insertion in the *lrpprc* genetic locus [94]. Zebrafish *lrpprc* mutants recapitulated the clinical phenotypes of Leigh Syndrome French-Canadian type such as altered mitochondrial gene expression, larval lethality, and defects in lipid homeostasis. Taking cues from the findings of these studies, these mutants do offer a significant potential to underpin the transcriptomic changes to the metabolic and bioenergetics readouts in the future.

The repository of mutants encoding for proteins involved in mitochondrial genome maintenance, respiratory chain biogenesis, assembly, and ion homeostasis offers the potential to understand the moonlighting role of these proteins. These models can help to decipher the role of these proteins in the mitochondrial interactome when studied in vivo. This underpins the utility of this MMC collection in deciphering the role of mitochondrial proteins in tissue-specific biological pathways. Taking the advantage of zebrafish as an excellent model system for therapeutic drug screening, our compendium of mutants provides an avenue to test many biological and chemical modulators as potential therapies for mitochondrial disorders for which treatment remains elusive.

These zebrafish mutants have been cryopreserved for the purpose of sharing the lines as a resource with the field for expanding the study of mitochondrial genetics and biology. In addition, our template for the creation of mitochondrial mutants in zebrafish should enable the creation of higher animal models of specific variants of interest. To accelerate this research, all GBT lines in this project had their information deposited with ZIRC, and all the lines were made available via zfishbook (http://www.zfishmeta.org/) [87]. The goal of this study is to disseminate research on the use of zebrafish in the field of mitochondrial biology and medicine, paving the way for the development of novel insights for diagnostic and therapeutic strategies. Ultimately, by using this collection of mutants we hope to unravel a small part of the mystery that shrouds one of the most crucial organelles in the cell. We hope that this MMC mutant collection and the primer we provide for adding to it will help to usher in new mitochondrial research using zebrafish.

## Figures and Tables

**Table 1:** List of nuclear-encoded genes for which zebrafish mutants were generated as part of MMC collection: The current MMC resource list summarizes information on the human gene, zebrafish ortholog, mouse ortholog, relevant clinical phenotypes and diseases, protein function, and OMIM ID. Allele designators are highlighted in bold for respective zebrafish paralog. (chr—Chromosome; OMIM ID—Online Mendelian Inheritance in Man ID; n/a—not available) *(mn1104gt and mn0582 are candidate lines*).

Approved Human Symbol	Approved Name	HUMAN chr	Zebrafish Orthologs	Zebrafish chr	Allele Designator	Mouse Orthologs	Mouse chr	Clinical Phenotype Observed in Humans	Disease	Biological Function	OMIM ID
**Mutants created by Transcription activator-like effector nucleases (TALEN)**											
*NDUFAF6*	NADH:ubiquinone oxidoreductase complex assembly factor 6	5	*ndufaf6*	16	**mn0117**	*Ndufaf6*	4	Focal right-hand seizures, ataxia, lactic acidosis, exercise intolerance, weakness, and muscle tension	Leigh syndrome, mitochondrial complex I deficiency	Oxidative phosphorylation complex assembly—Complex I	612392
*NDUFS4*	NADH: ubiquinone oxidoreductase subunit S4	5	*ndufs4*	5	**mn0118**	*Ndufs4*	13	Mitochondrial complex-I deficiency	Mitochondrial complex-I deficiency	Oxidative phosphorylation enzymes—Complex I	602694
*SDHA*	Succinate dehydrogenase complex flavoprotein subunit A	5	*sdha*	19	**mn0121**	*Sdha*	13	Dyspnea, cardiomegaly, cardiomyopathy, nystagmus, hypotonia, gastrointestinal stromal tumors, paragangliomas, pheochromocytoma, psychomotor regression and severe hyperandrogenism	Mitochondrial complex-II deficiency; Cardiomyopathy, Leigh syndrome; Paraganglioma	Oxidative phosphorylation enzymes—Complex II	600857
*COQ2*	Coenzyme Q2, polyprenyltransferase	4	*coq2*	5	**mn0106**	*Coq2*	5	Multiple system atrophy, unsteadiness of gait, nystagmus, gait ataxia, dysarthria, speech difficulty, dysmetria, lactic acidosis, urinary dysfunction, and nystagmus	Coenzyme Q10 deficiency, primary; Multiple system atrophy	Biosynthesis of CoQ, coenzyme in mitochondrial respiratory chain	609825
*PDSS2*	Decaprenyl-diphosphate synthase subunit 2	6	*pdss2*	13	**mn0120**	*Pdss2*	10	Hypotonia, seizures, cortical blindness, lactic acidosis, encephalopathy, and nephrotic syndrome	Coenzyme Q10 deficiency	Involved in biosynthesis of coenzyme Q	610564
*UQCRQ*	Ubiquinol- cytochrome c reductase complex III subunit VII	5	*uqcrq*	14	**mn0128**	*Uqcrq*	11	Severe psychomotor retardation and extrapyramidal signs, dystonia, athetosis, ataxia, mild axial hypotonia and marked global dementia	Mitochondrial complex III deficiency	Mitochondrial complex III: ubiquinol—cytochrome c reductase complex subunits	612080
*SURF1*	SURF1 cytochrome c oxidase assembly factor	9	*surf1*	5	**mn0123**	*Surf1*	2	Childhood onset neuropathy, lactic acidosis, mild intellectual disability ataxia, facial dysmorphism, encephalopathy, hypotonia, cerebellar ataxia, deafness, ophthalmoplegia, growth retardation and nystagmus	Charcot-Marie- Tooth disease, type 4K; Leigh syndrome, due to COX IV deficiency	Involved in biogenesis of cytochrome c oxidase complex	185620
*COX10*	Cytochrome c oxidase assembly factor heme A: farnesyltransferase COX10	17	*cox10*	12	**mn0107**	*Cox10*	11	Muscle weakness, hypotonia, ataxia, ptosis, pyramidal syndrome, status epilepticus, lactic acidosis, hypertrophic cardiomyopathy, hypoglycemia	Leigh syndrome due to mitochondrial COX IV deficiency; mitochondrial COX IV deficiency	Oxidative phosphorylation complex assembly—Complex IV	602125
*TMEM70*	Transmembrane protein 70	8	*tmem70*	2	**mn0126 mn0127**	*Tmem70*	1	Lactic acidosis, encephalopathy, histiocytoid cardiomyopathy, microcephaly, hypotonia, facial dysmorphism and 3- methylglutaconic aciduria psychomotor delay and hyperammonemia	Mitochondrial complex V (ATP synthase) deficiency	Biogenesis of mitochondrial ATP synthase	612418
*ATP5F1E*	ATP synthase F1 subunit epsilon	20	*atp5f1e*	6	**mn0101**	*Atp5f1e*	2	Neonatal-onset lactic acidosis, 3- methylglutaconic aciduria, mild mental retardation, and peripheral neuropathy	Mitochondrial complex (ATP synthase) deficiency	Oxidative phosphorylation complex assembly—ATP synthase	606153
*MCU*	Mitochondrial calcium uniporter	10	*mcu*	13	**mn0111**	*Mcu*	10	n/a	n/a	Calcium transporter and mediates calcium uptake in mitochondria	614197
*MICU1*	Mitochondrial calcium uptake 1	10	*micu1*	13	**mn0132**	*Micu1*	10	Proximal muscle weakness and learning disabilities	Myopathy with extrapyramidal signs	Regulator of mitochondrial calcium uptake	605084
*MTFMT*	Mitochondrial methionyl-tRNA formyltransferase	15	*mtfmt*	7	**mn0114** **mn0115**	*Mtfmt*	9	Psychomotor developmental delay, renal dysplasia, mild facial dysarthria and ataxia	Combined oxidative phosphorylation deficiency	Catalyzes the formylation of methionyl—tRNA	611766
**Mutants created by Gene-breaking transposon (GBT)**											
*MCU*	Mitochondrial calcium uniporter	10	*mcu*	13	**mn0599**	*Mcu*	10	n/a	n/a	Calcium transporter and mediates calcium uptake in mitochondria	614197
*LRPPRC*	Leucine rich pentatricopeptide repeat containing	2	*lrpprc*	13	**mn0235gt**	*Lrpprc*	17	Delayed psychomotor development, mental retardation, mild dysmorphic facial features, hypotonia, ataxia, development of lesions in the brainstem and basal ganglia, seizures, dysphagia, and hypertrophic cardiomyopathy	Leigh syndrome, French-Canadian type	Involved in translation of mitochondrial encoded cox subunits and mediation of folding of mitochondrial transcriptome	607544
*MRPS18B*	Mitochondrial ribosomal protein S18B	6	*mrps18b*	19	**mn0425gt**	*Mrps18b*	17	n/a	n/a	Part of small 28S subunit of mitoribosome	611982
*TIMM50*	Translocase of inner mitochondrial membrane 50	19	*timm50*	15	**mn0906gt**	*Timm50*	7	Severe intellectual disability, seizure and 3- methylglutaconic aciduria	3-methylglutaconic aciduria, type IX; Mitochondrial complex V deficiency	Subunit of TIM23 inner mitochondrial membrane complex and recognizes mitochondrial targeting signal or pre-sequence	607381
*OGDH*	Oxoglutarate dehydrogenase	7	*ogdhb*	10	**mn0281gt**	*Ogdh*	11	Colorectal cancer	Alpha- ketoglutarate dehydrogenase deficiency	Catalyzes the conversion of 2- oxoglutarate to succinyl-CoA and CO2	613022
*IDH2*	Isocitrate dehydrogenase (NADP (+)) 2, mitochondrial	15	*idh2*	18	**mn0268gt**	*Idh2*	7	Acute myeloid leukemia and abnormal production of D-2- hydroxyglutaric acid	D-2- hydroxyglutaric aciduria 2	Catalyzes the conversion of isocitrate to 2- oxoglutarate	147650
*CASQ1*	Calsequestrin	1	*casq1a*	2	**mn0437gt**	*Casq1*	1	Vacuolar myopathy	Myopathy, vacuolar, with CASQ1 aggregates	Luminal sarcoplasmic reticulum calcium sensor	114250
*DELE1*	DAP3 binding cell death enhancer 1	5	*dele1*	14	**mn0926gt**	*Dele1*	18	n/a	n/a	Key activator of the integrated stress response (ISR) following mitochondrial stress	615741
*NSUN2*	NOP2/Sun RNA methyltransferase 2	5	*nsun2*	19	mn1104gt*	*Nsun2*	13	Intellectual developmental disorder-5, some mild dysmorphic features, including microcephaly, long and narrow face, bushy eyebrows with synophrys, hypotelorism, large nose with long columella and hypoplastic alae nasi, short philtrum, and full upper lip, later onset of muscular hypertonia and spasticity	Intellectual developmental disorder, autosomal recessive 5	Methyltransferase that catalyzes the methylation of cytosine to 5-methylcytosine (m5C) at position 34 of intron-containing tRNA (Leu)(CAA) precursor	610916
LAP3*	Leucine aminopeptidase 3	4	*lap3*	1	mn0582gt*	*Lap3*	5	n/a	n/a	Enable peptidase activity	170250
*D2HGDH*	D-2-hydroxyglutarate dehydrogenase	2	*d2hgdh*	2	**mn1118gt**	*D2hgdh*	2	Tonic-clonic, and myoclonic seizures, D-2-hydroxyglutaric aciduria	D-2-hydroxyglutaric aciduria	Enables D-2hydroxyglutarate dehydrogenase, belonging to the FAD-binding oxidoreductase/transferase type 4 family	609186

## Data Availability

Raw RNA sequencing files have been uploaded on NCBI SRA: PRJNA861002.

## Supplementary Materials

The following supporting information can be downloaded at: https://www.mdpi.com/article/10.3390/genes13081317/s1: Figure S1: RNAseq of *coq2*, *uqcrq*, and *mcu* homozygous zebrafish mutant 6 dpf larvae. (**A**) Volcano plot of differentially expressed genes in the homozygous mutants. log_2_ of fold change and −log10 of *p*-value is represented on the x-axis and y-axis, respectively. The red dot signifies the differentially expressed genes with a *p*-value < 0.05 and the black dots represent the differentially expressed genes with a *p*-value of >0.05. Supplementary File S1: Genes associated with mitochondrial electron transport chain with zebrafish orthologs. Supplementary File S2: RNA sequencing data (differentially expressed genes and pathways) in *coq2* mutants. Supplementary File S3: RNA sequencing data (differentially expressed genes and pathways) in *uqcrq* mutants. Supplementary File S4: RNA sequencing data (differentially expressed genes and pathways) in *mcu* mutants.
