# Supplementary material for: A Primer Genetic Toolkit for Exploring Mitochondrial Biology and Disease Using Zebrafish
Source: Genes (Basel). 2022 Jul 23;13(8):1317. doi: 10.3390/genes13081317 (PMC9331066; doi:10.3390/genes13081317)
Supplement: Supplementary file 1 [file genes-13-01317-s001.zip › Supplementary figure s1.pdf]

Supplementary figure

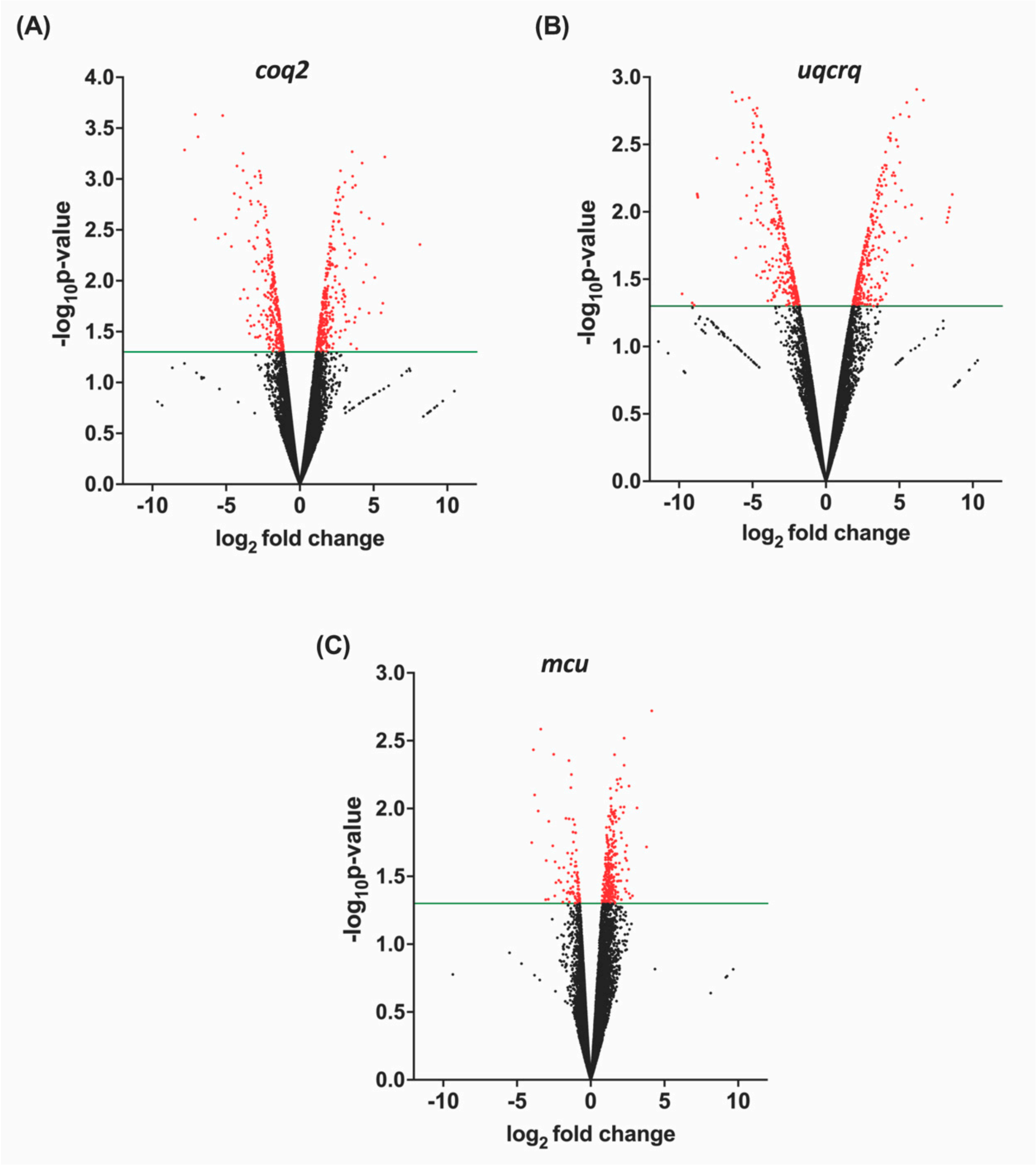

Supplementary figure S1: RNAseq of *coq2*, *uqcrq*, and *mcu* homozygous mutants (A–C). Volcano plot of differentially expressed genes in the homozygous mutants. log2 of fold change and −log10 of p-value is represented on the x-axis and y-axis, respectively. The red dot signifies the differentially expressed genes with a p-value<0.05 and the black dots represent the differentially expressed genes with a p-value of >=0.05.
